# New Possible Surgical Approaches for the Submammary Adipofascial Flap Based on Its Arterial Supply

**DOI:** 10.1155/2016/7696010

**Published:** 2016-09-29

**Authors:** Ehab M. Elzawawy, Melad N. Kelada, Ahmed F. Al Karmouty

**Affiliations:** ^1^Anatomy and Embryology, Faculty of Medicine, Alexandria University, Alexandria, Egypt; ^2^Surgery, Medical Research Institute, Alexandria University, Alexandria, Egypt

## Abstract

*Introduction*. Submammary adipofascial flap (SMAF) is a valuable option for replacement of the inferior portion of the breast. It is particularly useful for reconstruction of partial mastectomy defects. It is also used to cover breast implants. Most surgeons base this flap cranially on the submammary skin crease, reflecting it back onto the breast. The blood vessels supplying this flap are not well defined, and the harvest of the flap may be compromised due to its uncertain vascularity. The aim of the work was to identify perforator vessels supplying SMAF and define their origin, site, diameter, and length.* Materials and Methods*. The flap was designed and dissected on both sides in 10 female cadavers. SMAF outline was 10 cm in length and 7 cm in width. The flap was raised carefully from below upwards to identify the perforator vessels supplying it from all directions. These vessels were counted and the following measurements were taken using Vernier caliper: diameter, total length, length inside the flap, and distance below the submammary skin crease.* Conclusions*. The perforators at the lateral part of the flap took origin from the lateral thoracic, thoracodorsal, and intercostal vessels. They were significantly larger, longer, and of multiple origins than those on the medial part of the flap and this suggests that laterally based flaps will have better blood supply, better viability, and more promising prognosis. Both approaches, medially based and laterally based SMAF, carry a better prognosis and lesser chance for future fat necrosis than the classical cranially based flap.

## 1. Introduction

Breast cancer therapy includes mastectomy with breast reconstruction using implants or musculocutaneous flaps such as the transverse rectus abdominis musculocutaneous (TRAM) flap, latissimus dorsi flap, deep inferior epigastric perforator (DIEP) flap, and the superficial inferior epigastric artery (SIEA) flap [1–3].

The introduction of population-based breast screening programs has led to a higher proportion of women diagnosed with smaller cancers that are readily amenable to breast conserving therapy (BCT) [4].

BCT includes lumpectomy or skin-sparing partial mastectomy with immediate reconstruction using breast implants, local adipofascial flaps, or distant free dermal fat grafts. These techniques are called oncoplastic surgery. It allows cancer resection but prevents breast deformities by reconstructing defects immediately [5–7].

Jatoi and Proschan [8] concluded that mastectomy and BCT have comparable effects on mortality, even after long-term followup. Ogawa et al. [9] emphasized that BCT has become the standard strategy for breast cancer surgery ensuring local control with acceptable cosmetic results. Agarwal et al. [10] reported that patients who underwent BCT had a higher breast cancer-specific survival rate compared with those treated with mastectomy for early-stage invasive ductal carcinoma.

While many studies assessing musculocutaneous flaps and breast implants have been published, there is little data on breast reconstruction using adipofascial flaps. Immediate breast reconstruction with adipofascial flaps has become a safe option for patients with early-stage breast carcinoma [9, 11]. It can also be used to cover implants [12].

SMAF has a great importance in BCT. It occupies the area extending from the submammary crease cranially to the costal margin caudally and from the midline medially to the anterior axillary line laterally. Although it is frequently used, little is known about its vascular supply [11].

Defining the blood supply of this flap may give insight into the appropriate design of the flap, so immediate reconstruction with maximum viability is insured and incidence of flap necrosis is minimized.

## 2. Aim of the Work

The aim of the work was to identify perforator vessels supplying SMAF and define their origin, site, diameter, and length.

## 3. Material and Methods

**Figure 1:**
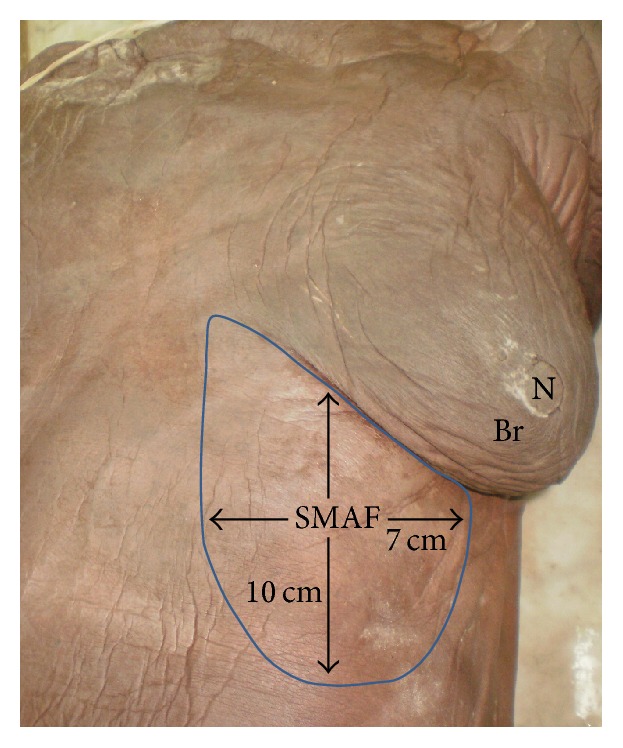
A photograph of left side of female cadaver showing the boundaries of submammary adipofascial flap (SMAF) marked on the skin just below the left breast (Br). Note that the length of the flap is about 10 cm while the width is about 7 cm, and N is the left nipple.

Ten female cadavers obtained from the dissecting room of the Anatomy Department, Faculty of Medicine, University of Alexandria, were included. SMAF was designed and dissected on both sides starting upwards at the submammary skin crease (upper border) and extending downwards for 10 cm (length) and starting medially 2 cm from midline and extending laterally for 7 cm (width) (Figure 1).

The flap was raised very carefully from below upwards to identify the perforator vessels supplying it from all directions. These vessels were counted and the following measurements were taken using Vernier caliper: diameter, total length, length inside the flap, and distance below the submammary skin crease. The data was collected and entered into the personal computer. Statistical analysis was done using Statistical Package for Social Sciences (SPSS/version 20) software. For comparison between groups, ANOVA test was used for parametric data, followed by post hoc test and Waller-Duncan method. The level of significance was 0.05. The same small letters indicate that there was no significant difference, while different letters indicate that there was a significant difference [13].

## 4. Results

### 4.1. Medial Perforators

#### 4.1.1. Perforators of the Superior Epigastric Vessels in the Lower Part

These vessels gave medial and lateral perforators. Lateral perforators passed into and supplied SMAF. They emerged through rectus abdominis muscle and rectus sheath. They ranged in number from 4 to 9 with a mean of 6.21 ± 1.42. They appeared on the surface at a mean distance of 1.28 ± 0.43 cm from midline.

They had a mean diameter of 1.31 ± 0.43 mm. They passed laterally for a mean total length of 5.51 ± 1.88. Their mean length inside the flap was 4.21 ± 2.36 cm (Tables 1 and 2). The upper 2 perforators were the longest reaching a maximal total length of 7.5 cm.

**Figure 2:**
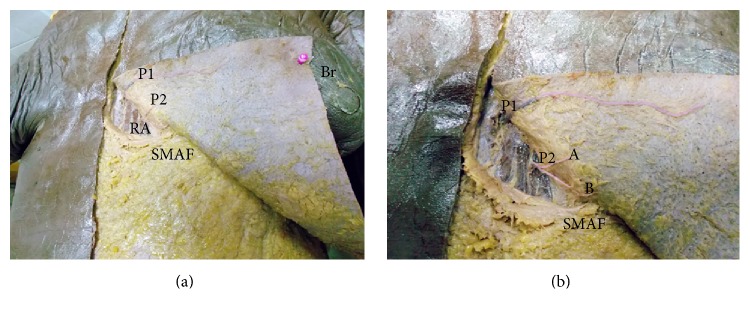
(a) A photograph of the left breast (Br) showing 2 perforators (P1, P2) arising from the superior epigastric artery and passing through rectus abdominis muscle (RA) to supply the upper medial part of SMAF. (b) A close-up photograph or the previous specimen showing P1 and P2. P1 is a long perforator supplying almost the whole width of the upper part of SMAF while P2 divides into 2 branches (A, B).

**Figure 3:**
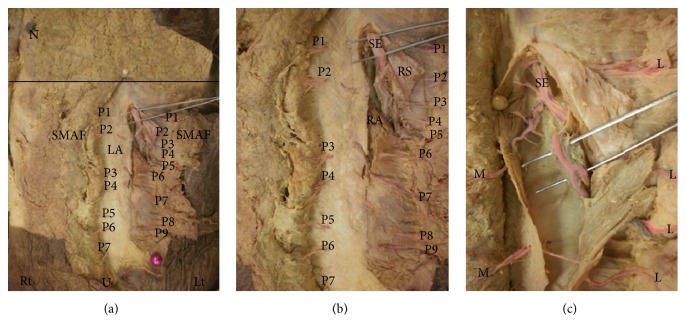
(a) A photograph of anterior abdominal wall showing 7 lateral perforators (P1→P7) on the right side (Rt) and 9 lateral perforators (P1→P9) on the left side (Lt). They supply SMAF. The right nipple (N), the umbilicus (U), and linea alba (LA) are noted. The submammary line is marked by dark line. Xiphoid process is marked by white pin. (b) A close-up photograph of the previous specimen. The perforators arise from the superior epigastric artery (SE) and pass through rectus abdominis muscle (RA) and rectus sheath (RS). (c) A close-up photograph showing the superior epigastric artery (SE) giving medial (M) and lateral (L) perforators. Lateral perforators pass into and supply SMAF.

These perforators were the most numerous but had smaller size and length than perforators supplying the flap from the lateral side (Table 3), so in spite of covering the whole length of SMAF, only the upper 2 perforators covered its whole width and can be used in the flap (Figures 2(a), 2(b), 3(a), 3(b), and 3(c)).

#### 4.1.2. Perforators of the Internal Mammary Vessels (IMV) in the Upper Part

**Figure 4:**
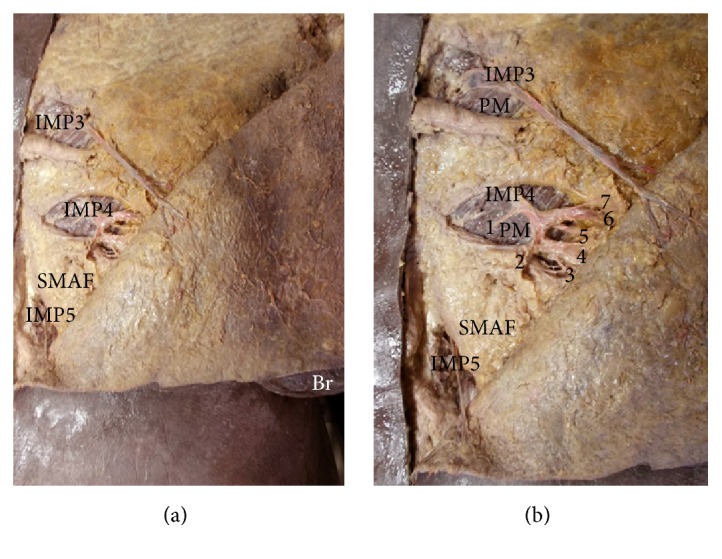
(a) A photograph of the left breast (Br) showing 3 perforators (IMP3, IMP4, and IMP5) arising from the internal mammary artery and supplying the upper medial part of SMAF. (b) A close-up photograph of the previous specimen. The perforators pass through pectoralis major muscle (PM) to supply the upper medial part of SMAF. IMP4 gives 7 branches (1–7) to SMAF, skin, and the mammary gland.

**Figure 5:**
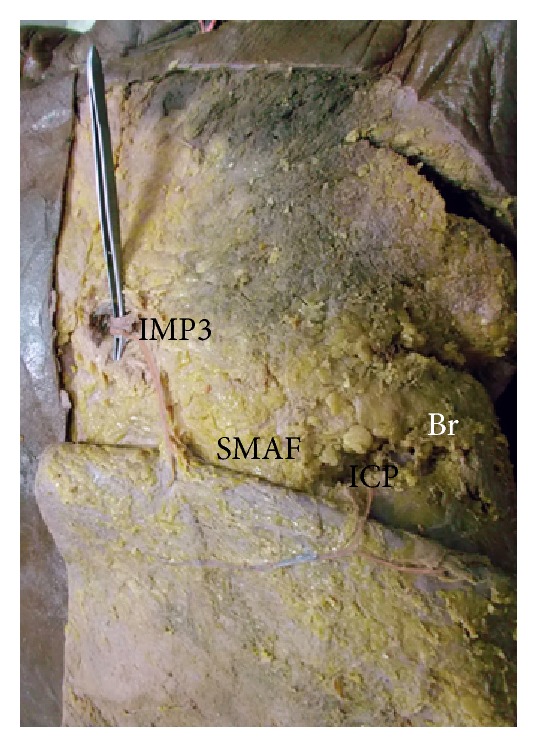
A photograph of the left breast (Br) showing 2 perforators: IMP3 to the medial part of SMAF and intercostal perforator (ICP) to the lateral part of SMAF.

**Figure 6:**
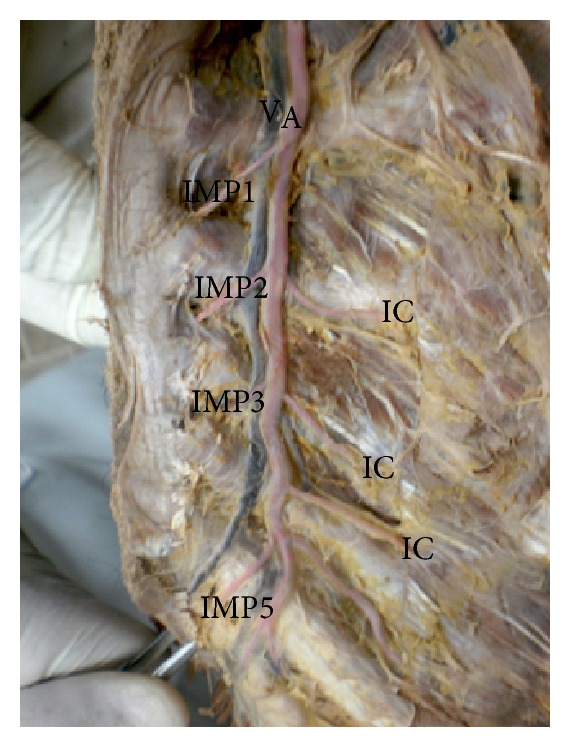
A photograph of opened anterior chest wall showing the internal mammary artery (A) and vein (V). The artery gives 4 perforators (IMP1, IMP2, IMP3, and IMP5) that pass through the anterior part of the intercostal spaces. It also gives anterior intercostal arteries (IC) passing laterally. IMP4 is missing.

Internal mammary perforators (IMPs 3, 4, and 5) passed through the anterior parts of the intercostal spaces and the sternal origin of pectoralis major muscle to supply the flap, and their number ranged from 2 to 3 with a mean of 2.5 ± 0.66. They appeared on the surface at a mean distance of 1.8 ± 0.25 cm from the midline. They had a mean diameter of 1.66 ± 0.53 mm and mean total length of 5.24 ± 1.12. Their mean length inside the flap was 3.44 ± 1.12 cm. They were sizeable but did not cover the whole width or length of the flap; they only covered the upper medial part of SMAF (Figures 4(a), 4(b), 5, and 6) (Tables 1 and 2). IMP4 is especially large in females and is the main internal mammary perforator supplying SMAF. It reached up to 6.5 cm in length and 2.2 mm in diameter. It was absent in one case on one side (Figure 6).

### 4.2. Lateral Perforators

#### 4.2.1. Perforators of the Thoracodorsal Vessels (TDV): Two Types

**Figure 7:**
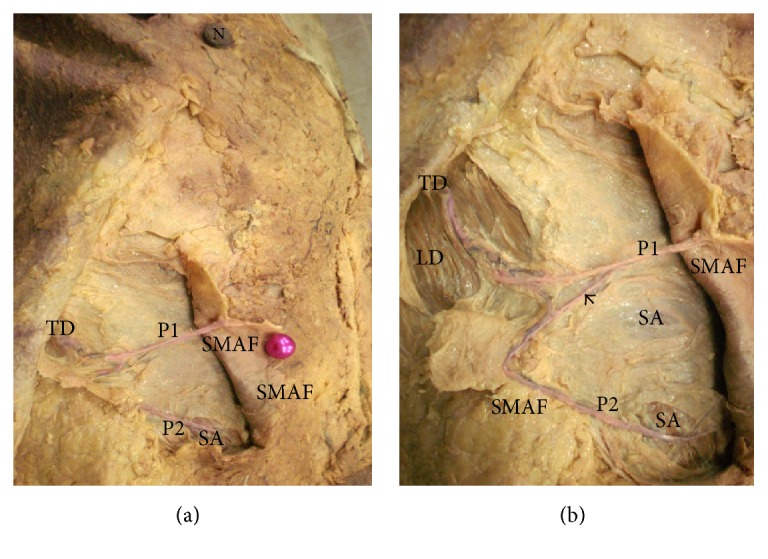
(a) A photograph of the right breast showing the distal part of thoracodorsal artery (TD) giving a perforator (P1) that passes directly to supply the upper lateral part of SMAF. TD also gives P2 that supplies serratus anterior muscle (SA). The right nipple is noted (N). (b) A close-up photograph of the previous specimen showing TD passing through latissimus dorsi muscle (LD). P2 supplies serratus anterior muscle (SA) and passes to the lower lateral part of SMAF. There is a connecting loop between the 2 perforators (arrow).

**Figure 8:**
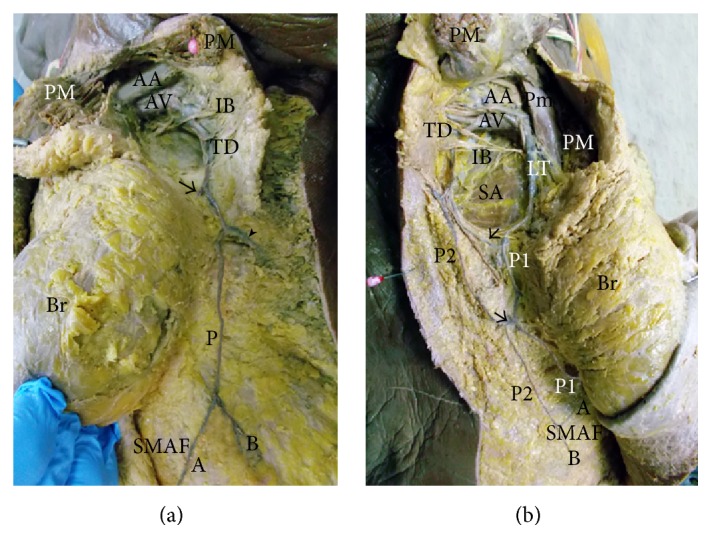
(a) A photograph of left breast (Br) showing the pectoralis major muscle (PM) cut and reflected to show the axillary artery (AA) and axillary vein (AV) giving the thoracodorsal vessels (TD) that run downwards and give a branch (arrow) to the left breast and the circumflex scapular vessels (arrow head) and terminate as perforator (P) to SMAF. This perforator divides into medial (A) and lateral (B) branches. The intercostobrachial nerve (IB) is noted. (b) A photograph of the right breast (Br) showing the pectoralis major muscle (PM) cut and reflected and the pectoralis minor muscle (Pm) deep to it. The axillary artery (AA) and axillary vein (AV) can be seen. They give the lateral thoracic vessels (LT) which supply the breast itself and give perforator (P1) to SMAF. They also give the thoracodorsal vessels (TD) which give perforator (P2) that communicates through branches (arrows) with P1 and supply SMAF. The distal part of P2 gives 2 capillary perforators, upper (A) and lower (B). Serratus anterior muscle (SA) can be seen. The intercostobrachial nerve (IB) is noted.

**Figure 9:**
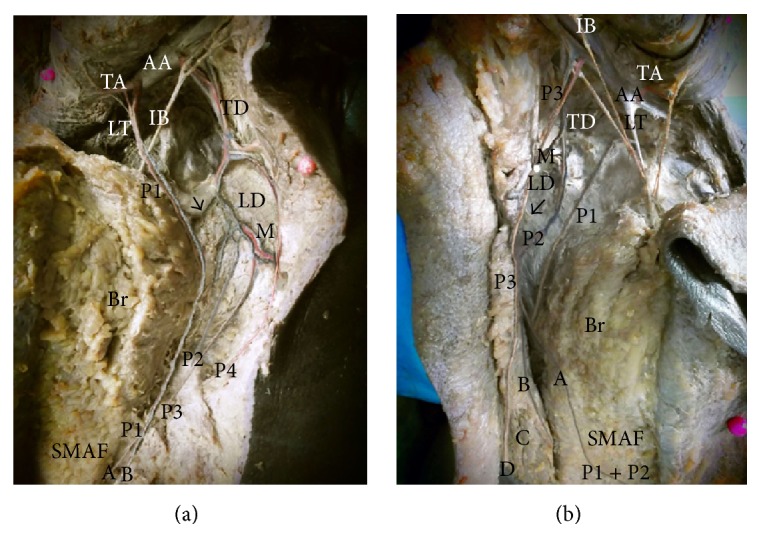
(a) A photograph of the left breast (Br) showing the axillary artery (AA) giving the long thoracic artery (LT) which gives perforator (P1) to SMAF and the thoracodorsal artery (TD) which gives muscular branch (M) to latissimus dorsi (LD) and 3 perforators (P2, P3, and P4) to SMAF. A communicating vessel between P1 and P2 is pointed by arrow. P1 gives 2 capillary perforators: upper (A) and lower (B) in SMAF. Thoracoacromial artery (TA) and intercostobrachial nerve (IB) are noted. (b) A photograph of the right breast (Br) showing the axillary artery (AA) giving thoracoacromial artery (TA), long thoracic artery (LT), and thoracodorsal artery (TD). LT gives perforator (P1). TD gives muscular branch (M) to latissimus dorsi (LD) and perforator (P2). P2 divides into medial branch (A) and lateral branch (B). P1 and (A) join and supply SMAF. AA gives a direct perforator (P3) to SMAF. P3 divides into medial branch (C) and lateral branch (D). A communicating vessel between P2 and P3 is pointed by arrow. The intercostobrachial nerve (IB) is noted.

(I) Serratus anterior collaterals: they passed through and supplied serratus anterior muscle and terminated in the lateral part of the flap (Figures 7(a) and 7(b)).
(II) Cutaneous branches of the thoracodorsal artery: they passed just anterior to the lateral edge of latissimus dorsi parallel and posterior to the lateral thoracic artery with which they had several communications. They gave medial and lateral or upper and lower terminal branches covering the whole lateral part of the flap (Figures 8(a), 8(b), 9(a), and 9(b)).
 Both types of perforators were lengthy and sizable. Their number ranged from 2 to 4 (mean 2.88 ± 0.92). Their mean diameter was 2.11 ± 0.25 mm and their mean total length was 10 ± 2.21 cm while their mean length inside the flap was 5.13 ± 1.50 cm (Tables 1 and 2). They gave capillary perforators that passed into the flap for few centimeters and had a diameter of about 0.5 mm (Figures 8(b) and 9(a)).

#### 4.2.2. Perforators of the Lateral Thoracic Vessels

Their number ranged from 1 to 3 (mean 2.15 ± 0.61) and they supplied the upper lateral part of the flap. Their mean diameter was 1.82 ± 0.44 mm and their mean total length was 10.52 ± 3.1 cm while their mean length inside the flap was 5.42 ± 0.93 cm. They were long and sizeable perforators (Figures 8(b), 9(a), and 9(b)) (Tables 1 and 2). A direct perforator may arise from the axillary artery and supply SMAF (Figure 9(b)).

#### 4.2.3. Perforators of the Intercostal Vessels

**Figure 10:**
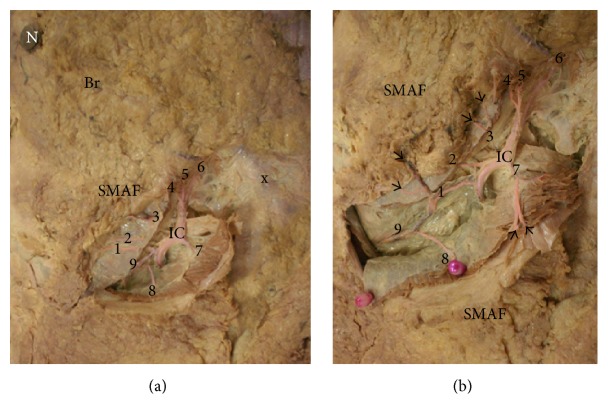
(a) A photograph of the right breast (Br) showing 9 perforators (1→9) supplying SMAF. They arise from the lateral cutaneous branch of the intercostal artery (IC). Right nipple (N) and the xiphoid process (x) are noted. (b) A close-up photograph of the previous specimen showing that each perforator branches into smaller capillary perforators (arrows).

These are the anterior perforators of the lateral cutaneous branches of the posterior intercostal arteries. They passed through the intercostal muscles and serratus anterior muscle. They appeared in the lateral half of the flap and supplied mainly its lower lateral part. Their number ranged from 2 to 4 with a mean of 2.91 ± 0.89 and their mean diameter was 1.22 ± 0.31 mm. Their mean total length was 4.16 ± 1.38 cm while their mean length inside the flap was 2.81 ± 1.03 cm. A large perforator was found in the 6th or 7th intercostal space having a length of 6 cm and a diameter of 1.5 mm. It can give up to 9 perforators which may branch into smaller capillary perforators in the flap (Figures 5, 10(a), and 10(b)) (Tables 1 and 2).

## 5. Discussion

For many years, breast reconstruction focused on utilizing muscle, fascia, fat, and skin to reconstruct the defect. Recently, a whole new concept of breast reconstruction using local fat and fascia only without any muscular or cutaneous compromise was introduced.

Pearl and Johnson [14] demonstrated an extensive continuous subcutaneous vascular network existing between the dense and loose adipose tissue. This was an important innovation in reconstructive plastic surgery. It provides the basis for adipofascial flaps. However, these flaps cannot be used in cases of extensive and complex loss of tissue, for which a microvascular myocutaneous flap is always the best solution [15].

The latissimus dorsi myocutaneous flap is usually the best choice for defects that are too large to be corrected by adipofascial flaps. However, this technique must be performed by plastic surgeons and involves the loss of donor-site muscle and skin [2].

The location of the tumor significantly influences cosmetic outcome with worse results for inferior locations especially when postoperative radiotherapy is used [16]. Ogawa et al. [9] stated that it is more difficult to repair defects in the lower portion of the breast than defects in the upper portion of the breast.

SMAF used in this study is located very close to the inferior portion of the breast, and this procedure is easy to perform, thus making it possible for breast surgeons to perform it without the help of plastic surgeons. SMAF is supplied by local perforators from multiple sources and avoids the sacrifice of muscles, skin, and large vascular pedicles.

Kijima et al. [11] developed an adipofascial flap from the anterior sheath of the rectus abdominis and fat in front of it. Even if the patient is not obese, a large amount of fat can be obtained for reconstruction of lower breast defects.

Aljarrah et al. [17] recommend the use of a crescent SMAF to fill the cavity resulting from the excision of tumors situated near the inframammary fold. They recommend its use as a first step oncoplastic surgery technique in patients with small or medium sized breasts. They concluded that the technique can be performed quickly and does not require any contralateral symmetrization. Forty five months of mean followup showed satisfactory cosmetic results.

Otsuka et al. [18] performed inframammary adipofascial tissue repair (IATR) on 25 patients with breast cancer in inferior site. They used a tongue shaped flap to repair the defect. There was no partial or total necrosis of the flap 1 year after surgery.

Kijima et al. [7] used free dermal fat graft for breast reconstruction. They reported a gradually diminished size of the graft. SMAF has a great advantage over free dermal fat graft because in SMAF fat is transposed upwards with its blood supply.

These techniques achieve better cosmetic results than the transposition of residual breast tissue and are more convenient than muscle flap grafting and safer than implantation of foreign materials. Immediate volume replacement and softness of the breast were obtained without fat necrosis or contour irregularity. However, insufficient resection margins that occur from paying too much attention to cosmetics may increase local recurrence.

Regarding perforators number in the present study, superior epigastric vessel had the largest number, followed by the other vessels. However, the upper 2 superior epigastric perforators are the ones that can be easily incorporated in the flap due to their length. The lower superior epigastric perforators are short and therefore cannot be used in the flap. The lateral thoracic and thoracodorsal perforators were the longest followed by other perforators. They had the greatest length inside the flap together with the upper 2 superior epigastric perforators. They had the greatest diameter together with the internal mammary perforators (Table 3).

Based on these results, it can be assumed that the design of the flap with the base superiorly oriented at the submammary crease has the least secure blood supply. In this situation the flap is based on one or at maximum two perforator vessels of the internal mammary artery (2.03 ± 1.1 cm from submammary crease). Although this design is frequently used, the viability is endangered and flap fat necrosis at long term is likely.

On the other hand, basing the flap on its medial part towards midline appears to be more appropriate. In this situation, large perforators of the superior epigastric and internal mammary vessels supply extended length of the flap. So preservation of these large perforators at the base enables harvest of extended portion of the flap which can be rotated cranially to replace medial and inferior portions of the breast.

Similarly, when SMAF is based laterally towards the anterior axillary line, a raw of large vessels supplies extended portion of the flap. This raw of vessels begins cranially as perforators of the lateral thoracic, followed by perforators of the thoracodorsal, and then the perforators of the intercostal vessels more caudally. These perforators are lengthy and have a large caliber. This flap has a large rotation arc due to its lengthy perforators and can be designed with the base oriented laterally, rotated cranially to replace central, inferior, and lateral portions of the breast.

Masetti et al. [19] concluded that lateral rotation flaps are very useful for tumors located in the lateral portion of the breast and cannot be used for defects in the inferior portion because they are too far. However, the results of the present work prove that lateral perforators are indeed long enough to cover the inferior portion of the breast.

These approaches are not frequently used and give more importance and indications for SMAF rather than the classical cranially based flap that is frequently used to replace the inferior portion of the breast.

Traditionally, in large defects following radical mastectomy, innominate vessels (IMV) or thoracodorsal vessels (TDV) are used as recipient vessels for TRAM and DIEP flaps [20].

Nevertheless, the use of IMV itself carries several risks such as pneumothorax, parasternal or intercostal hernia, and intercostal neuralgia [21]. A case of intraoperative cardiac tamponade and fatal bleeding related to IMV use was reported [22]. Finally, the use of IMV for breast reconstruction renders them unavailable for aortocoronary bypass [23].

The use of IMPs (internal mammary perforators) has demonstrated success and avoids the complications related to the use of IMV itself. These vessels are easy to expose and have suitable caliber [24].

Munhoz [24] found that in immediate reconstruction these perforators are present in 93.5%; their presence decreases to 12.9% in delayed reconstruction. In the present work, IMPs supplying SMAF ranged from 2 to 3 in number, their mean length was 5.24 ± 1.12 cm, their mean diameter was 1.66 ± 0.53 mm, and they were found in most cases giving great importance for medially based SMAF. Only one case had no IMP4 on one side.

Pompeo et al. [25] reported that IMPs presence is dependent on anatomical variability. Tan et al. [26] proposed Multidetector CT Angiography, as a noninvasive imaging method to document the anatomic characteristics of IMPs. Schmidt et al. [27] reported seven out of 100 dissected intercostal spaces with no IMPs.

IMPs 3, 4, and 5 run superficially in the subcutaneous tissue in a laterocaudal direction to supply SMAF. However, the use of flaps based on the IMPs has rarely been reported. The results of Schmidt et al. [27] indicate that IMPs flaps can be raised from an average of 13 × 7 cm up to the size of 20 × 13 cm. This is comparable to the lateral intercostal artery perforator (LICAP) flaps (average 18 × 8 cm; up to the size of 24 × 12 cm) [28]. The present study agrees with Schmidt et al. [27] that flaps based on IMPs 3 and 4 in women would be thicker, containing a bigger amount of subcutaneous fat, and can be used for breast reconstruction.

Pompeo et al. [29] prefer TDV over IMV as recipient vessels for myocutaneous flaps because of their greater and more constant caliber and the feasibility of immediate reconstruction due to the pedicle's length. However, the use of thoracodorsal artery perforator (TAP) avoids the sacrifice of the entire TDV pedicle and assures the vitality of the myocutaneous latissimus dorsi flap, useful for secondary salvage reconstructive procedure [30].

J. T. Kim and S. W. Kim [30] classified three types of TAP flaps, namely, transmuscular, fasciocutaneous, and direct cutaneous perforator flaps. They can be used for reconstruction of the extremities, for head and neck as free style flaps, and as pedicled flaps for reconstruction of the chest wall and axillary wounds. Mobilizing local fat and fascia with their fasciocutaneous perforators does not compromise skin which has direct cutaneous perforators from several sources [31].

TAP flap with capillary perforators (TAPcp) is based on the fact that small capillary perforators arising from the descending branch of the thoracodorsal artery can nourish a large area of skin and subcutaneous fat. The diameters of these capillary perforators are approximately 0.3 to 0.5 mm. In the present work, large TAPs were spotted with mean diameter of 2.11 ± 0.25 mm and mean total length of 10 ± 2.21 cm and their number ranged from 2 to 4. They gave capillary perforators inside SMAF that had a diameter of about 0.5 mm. These capillary perforators can be located with great precision using preoperative color Doppler US [32].

The presence of TAPs is consistent and the length of perforators in itself is good enough. TAP flap has a versatile utility and surgical ease of harvest and anastomosis [33].

Direct cutaneous and fasciocutaneous perforators arise from the lateral thoracic artery or directly from the axillary artery and run vertically down the lateral chest wall. These perforators give smaller perpendicular capillary perforators towards the skin and subcutaneous fat, which are the vascular basis of the LTAP (lateral thoracic artery perforator) flap [34].

The present study considers LTAP as a main blood supply for SMAF because of their large caliber (1.82 ± 0.44 mm), long pedicle (10.52 ± 3.1 cm), and great length inside SMAF (5.42 ± 0.93 cm). LTAP tend to be positioned more superiorly and medially than TAP and LICAP flaps, making it very simple to incorporate them in a laterally based SMAF.

The initial work of McCulley et al. [35] with lateral breast reconstruction focused on using the LICAP flap for immediate volume replacement and either LICAP or TAP flap for delayed reconstruction. As their use of these lateral chest wall techniques increased, they regularly noticed good lateral thoracic artery perforators (LTAP) and developed experience in using LTAP flap. It is their current practice to use LTAP and LICAP flaps predominantly in the immediate reconstructive setting. These flaps do not sacrifice any main pedicle, are considered “expendable,” and do not compromise any major reconstruction options in cases where the margins are found to be incomplete and mastectomy is required.

The present results agree with Hamdi et al. [28] who described LICAP flaps and reported that they can provide reconstructive options for defects in the chest wall. They described a reliable dominant lateral perforator in the 5th to 8th intercostal spaces. Its diameter reaches up to 1.5 mm and its length is 5-6 cm.

Hamdi et al. [36] consider LICAP flaps as the first choice for reconstruction of lateral breast defects, whereas TAP flap is a better reconstructive option for central defects of the breast.

Salim and Chana [37] used LICAP flap as an adipofascial flap to correct complication of gynaecomastia over-excision. They advocate its use as a fasciocutaneous flap for breast reconstruction.

**Figure 11:**
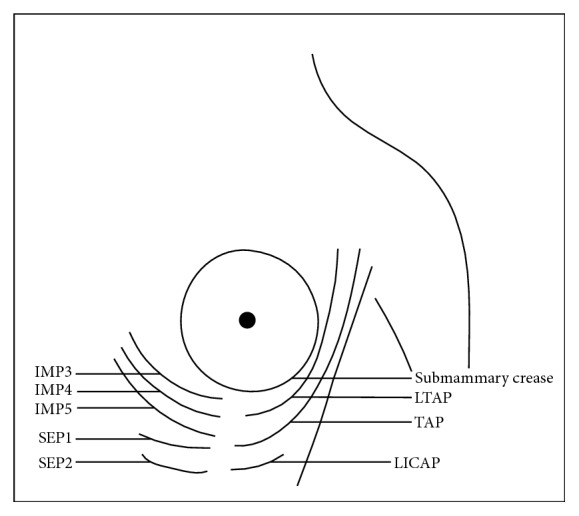
Diagrammatic illustration of the perforators supplying SMAF. LTAP: lateral thoracic artery perforator. TAP: thoracodorsal artery perforator. LICAP: lateral intercostal artery perforator. IMP3: internal mammary perforator 3. IMP4: internal mammary perforator 4. IMP5: internal mammary perforator 5. SEP1: superior epigastric perforator 1. SEP2: superior epigastric perforator 2.

The algorithm described by Levine et al. [38] for lateral breast reconstruction recommends that dissection should be performed from lateral to medial, making TAP the first option, then moving medially onto LTAP and LICAP vessels as suitable. The present study suggests that dissections should start downwards and advance superiorly to incorporate all possible perforators in the flap. In doing so, the LICAP are met first (5.41 ± 1.34 cm from submammary crease) followed by TAP at 4.61 ± 1.26 cm and the highest are LTAP at 3.88 ± 1.06 cm (Figure 11) (Table 3).

SMAF is a perforator flap and includes LICAP flap [39], LTAP flap [40], and TAP flap [33]. Using all three potential sources of perforators allows a greater degree of versatility in lateral breast reconstructions.

## 6. Conclusion

Both approaches medially based and laterally based SMAF carry a better prognosis and lesser chance for future fat necrosis than the classical cranially based flap. While perforators entering at the medial border of SMAF are significantly more numerous than those at the lateral border, the perforators at the lateral part of the flap are significantly larger, longer, and of multiple origins. This suggests that laterally based flaps will have better viability and better chance for survival.

## Figures and Tables

**Table 1:** Number, total length, and diameter of perforators supplying SMAF.

Perforator	Number			Total length			Diameter		
	Mean ± SD	Min.	Max.	Mean ± SD	Min.	Max.	Mean ± SD	Min.	Max.
Superior epigastric	6.21 ± 1.42	4	9	5.51 ± 1.88	4.2	7.5	1.31 ± 0.43	0.91	1.6
Internal mammary	2.50 ± 0.66	2	3	5.24 ± 1.12	3.7	6.5	1.66 ± 0.53	1.1	2.2
Thoracodorsal	2.88 ± 0.92	2	4	10 ± 2.21	8.5	13	2.11 ± 0.25	1.9	2.3
Lateral thoracic	2.15 ± 0.61	1	3	10.52 ± 3.1	7.4	14	1.82 ± 0.44	1.5	2.2
Intercostals	2.91 ± 0.89	2	4	4.16 ± 1.38	3.5	6	1.22 ± 0.31	1	1.5

**Table 2:** Site of different perforators indicated by length inside SMAF and distance from submammary skin crease.

Perforator	Length inside SMAF (cm)			Distance from submammary skin crease (cm)		
	Mean ± SD	Min.	Max.	Mean ± SD	Min.	Max.
Superior epigastric	4.21 ± 2.36	2.91	6.6	6.5 ± 3.1	3.22	10
Internal mammary	3.44 ± 1.12	2.23	4.7	2.03 ± 1.1	1.06	3.0
Thoracodorsal	5.13 ± 1.50	3.32	6.8	4.61 ± 1.26	3.4	5.9
Lateral thoracic	5.42 ± 0.93	4.14	6.5	3.88 ± 1.06	2.9	4.9
Intercostals	2.81 ± 1.03	2.46	4.12	5.41 ± 1.34	4.0	6.8

For the superior epigastric and internal mammary vessels, length inside SMAF was measured as they entered its medial border, while for the thoracodorsal, lateral thoracic, and anterior perforators of the intercostal vessels the length was measured as they entered the lateral border of the flap.

**Table 3:** Comparison between medial and lateral perforators.

Perforator	Medial perforators		Lateral perforators			*P*
	Superior epigastric	Internal mammary	Thoracodorsal	Lateral thoracic	Intercostals	
Number	6.21 ± 1.44^a^	2.50 ± 0.66^b^	2.88 ± 0.92^b^	2.15 ± 0.61^b^	2.91 ± 0.89^b^	0.011^*∗*^
Total length	5.51 ± 1.88^b^	5.24 ± 1.12^b^	10.0 ± 2.21^a^	10.52 ± 3.1^a^	4.16 ± 1.38^b^	0.0136^*∗*^
Diameter	1.31 ± 0.43^b^	1.66 ± 0.53^ab^	2.11 ± 0.25^a^	1.82 ± 0.44^a^	1.22 ± 0.31^b^	0.042^*∗*^
Length inside flap	4.21 ± 2.36^b^	3.44 ± 1.12^b^	5.13 ± 1.50^a^	5.42 ± 0.93^a^	2.81 ± 1.03^c^	0.033^*∗*^
Distance from submammary crease	6.5 ± 3.1^a^	2.03 ± 1.1^c^	4.61 ± 1.26^b^	3.88 ± 1.06^bc^	5.41 ± 1.34^b^	0.027^*∗*^

The same small letters indicate that there was no significant difference between groups while different letters indicate significant difference. *∗* means significant difference.
